# Supplementary material for: The association between BCG treatment in patients with bladder cancer and subsequent risk of developing Alzheimer and other dementia.—A Swedish nationwide cohort study from 1997 to 2019
Source: PLoS One. 2023 Dec 14;18(12):e0292174. doi: 10.1371/journal.pone.0292174 (PMC10721016; doi:10.1371/journal.pone.0292174)
Supplement: S1 Table — (DOCX) [file pone.0292174.s001.docx]

## S1 Table. Subgroup analyses of patients follow-up in relevant age.

###

### Alzheimer’s

Median follow-up time: 5.1 years

|  |  |  | **Univariate** | | | **Multivariate** | | |
| --- | --- | --- | --- | --- | --- | --- | --- | --- |
|  |  | **N** | **HR** | **CI95%** | **P-value** | **HR** | **CI95%** | **P-value** |
| BCG | No BCG | 10804 | 1 | - | - | 1 | - | - |
|  | BCG | 1994 | 0.86 | 0.64-1.17 | 0.339 | 0.87 | 0.62-1.23 | 0.430 |
| T stage | Ta | 8260 | 1 | - | - | 1 | - | - |
|  | Tis | 452 | 0.64 | 0.33-1.24 | 0.181 | 0.70 | 0.35-1.41 | 0.321 |
|  | T1 | 4086 | 1.06 | 0.85-1.33 | 0.615 | 1.07 | 0.83-1.39 | 0.587 |
|  |  |  | P(overall): 0.285 | | | P(overall): 0.426 | | |
| Grade | G1/LMP/<NA> | 4185 | 1 | - | - | 1 | - | - |
|  | G2 | 5375 | 1.06 | 0.84-1.33 | 0.616 | 1.07 | 0.84-1.36 | 0.585 |
|  | G3-G4/anaplastic | 3238 | 1.01 | 0.76-1.34 | 0.953 | 1.05 | 0.74-1.48 | 0.793 |
|  |  |  | P(overall): 0.866 | | | P(overall): 0.862 | | |
| Sex | M | 9654 | 1 | - | - | 1 | - | - |
|  | F | 3144 | 0.96 | 0.76-1.21 | 0.720 | 0.95 | 0.75-1.21 | 0.697 |
| Age | <=75 | 3326 | 1 | - | - | 1 | - | - |
|  | >75 | 9472 | 1.69 | 1.34-2.13 | <0.001 | 1.65 | 1.31-2.09 | <0.001 |
| CCI | No comorbidity (0) | 6010 | 1 | - | - | 1 | - | - |
|  | Mild comorbidity (1) | 2489 | 1.25 | 0.95-1.65 | 0.104 | 1.22 | 0.93-1.60 | 0.159 |
|  | Intermediate comorbidity (2) | 2387 | 1.05 | 0.77-1.43 | 0.781 | 1.00 | 0.73-1.36 | 0.985 |
|  | Severe comorbidity (>2) | 1912 | 1.71 | 1.25-2.35 | <0.001 | 1.61 | 1.17-2.21 | 0.004 |
|  |  |  | P(overall): 0.011 | | | P(overall): 0.029 | | |

### Dementia

Median follow-up time: 4.9 years

|  |  |  | **Univariate** | | | **Multivariate** | | |
| --- | --- | --- | --- | --- | --- | --- | --- | --- |
|  |  | **N** | **HR** | **CI95%** | **P-value** | **HR** | **CI95%** | **P-value** |
| BCG | No BCG | 10804 | 1 | - | - | 1 | - | - |
|  | BCG | 1994 | 0.91 | 0.79-1.05 | 0.191 | 0.81 | 0.69-0.95 | 0.010 |
| T stage | Ta | 8260 | 1 | - | - | 1 | - | - |
|  | Tis | 452 | 0.87 | 0.67-1.15 | 0.335 | 0.93 | 0.70-1.24 | 0.627 |
|  | T1 | 4086 | 1.14 | 1.03-1.27 | 0.015 | 1.11 | 0.98-1.26 | 0.087 |
|  |  |  | P(overall): 0.024 | | | P(overall): 0.153 | | |
| Grade | G1/LMP/<NA> | 4185 | 1 | - | - | 1 | - | - |
|  | G2 | 5375 | 1.00 | 0.89-1.12 | 0.975 | 1.02 | 0.91-1.15 | 0.690 |
|  | G3-G4/anaplastic | 3238 | 1.17 | 1.03-1.33 | 0.017 | 1.21 | 1.04-1.42 | 0.017 |
|  |  |  | P(overall): 0.028 | | | P(overall): 0.039 | | |
| Sex | M | 9654 | 1 | - | - | 1 | - | - |
|  | F | 3144 | 1.01 | 0.90-1.12 | 0.909 | 1.00 | 0.90-1.12 | 0.979 |
| Age | <=75 | 3326 | 1 | - | - | 1 | - | - |
|  | >75 | 9472 | 2.38 | 2.12-2.68 | <0.001 | 2.30 | 2.05-2.59 | <0.001 |
| CCI | No comorbidity (0) | 6010 | 1 | - | - | 1 | - | - |
|  | Mild comorbidity (1) | 2489 | 1.31 | 1.15-1.50 | <0.001 | 1.26 | 1.10-1.44 | <0.001 |
|  | Intermediate comorbidity (2) | 2387 | 1.29 | 1.12-1.49 | <0.001 | 1.20 | 1.05-1.38 | 0.010 |
|  | Severe comorbidity (>2) | 1912 | 1.89 | 1.63-2.20 | <0.001 | 1.74 | 1.49-2.02 | <0.001 |
|  |  |  | P(overall): <0.001 | | | P(overall): <0.001 | | |
