# Supplementary material for: The association between BCG treatment in patients with bladder cancer and subsequent risk of developing Alzheimer and other dementia.—A Swedish nationwide cohort study from 1997 to 2019
Source: PLoS One. 2023 Dec 14;18(12):e0292174. doi: 10.1371/journal.pone.0292174 (PMC10721016; doi:10.1371/journal.pone.0292174)
Supplement: S2 Table — (DOCX) [file pone.0292174.s002.docx]

**S2 Table.** **Multivariate Cox analysis of risk of dementia and AD between BCG treated and non BCG treated patients**

### Alzheimer’s

|  |  |  | **Univariate** | | | **Multivariate** | | |
| --- | --- | --- | --- | --- | --- | --- | --- | --- |
|  |  | **N** | **HR** | **CI95%** | **P-value** | **HR** | **CI95%** | **P-value** |
| BCG | No BCG | 23597 | 1 | - | - | 1 | - | - |
|  | BCG | 6060 | 1.01 | 0.81-1.27 | 0.912 | 0.98 | 0.75-1.27 | 0.854 |
| T stage | Ta | 20543 | 1 | - | - | 1 | - | - |
|  | Tis | 1043 | 1.00 | 0.63-1.61 | 0.987 | 0.96 | 0.58-1.59 | 0.865 |
|  | T1 | 8071 | 1.23 | 1.02-1.49 | 0.032 | 1.08 | 0.87-1.35 | 0.479 |
|  |  |  | P(overall): 0.106 | | | P(overall): 0.730 | | |
| Grade | G1/LMP/<NA> | 11178 | 1 | - | - | 1 | - | - |
|  | G2 | 11564 | 1.23 | 1.02-1.49 | 0.034 | 1.16 | 0.95-1.41 | 0.151 |
|  | G3-G4/anaplastic | 6915 | 1.34 | 1.07-1.69 | 0.012 | 1.20 | 0.90-1.59 | 0.214 |
|  |  |  | P(overall): 0.022 | | | P(overall): 0.293 | | |
| Sex | M | 22434 | 1 | - | - | 1 | - | - |
|  | F | 7223 | 1.09 | 0.90-1.31 | 0.389 | 1.09 | 0.90-1.32 | 0.370 |
| Age | <=75 | 18028 | 1 | - | - | 1 | - | - |
|  | >75 | 11629 | 3.34 | 2.80-4.00 | <0.001 | 3.11 | 2.59-3.73 | <0.001 |
| CCI | No comorbidity (0) | 16831 | 1 | - | - | 1 | - | - |
|  | Mild comorbidity (1) | 4790 | 1.59 | 1.26-1.99 | <0.001 | 1.36 | 1.08-1.71 | 0.009 |
|  | Intermediate comorbidity (2) | 4748 | 1.23 | 0.94-1.60 | 0.128 | 1.03 | 0.78-1.34 | 0.849 |
|  | Severe comorbidity (>2) | 3288 | 2.28 | 1.73-2.99 | <0.001 | 1.75 | 1.32-2.31 | <0.001 |
|  |  |  | P(overall): <0.001 | | | P(overall): <0.001 | | |

### Dementia

|  |  |  | **Univariate** | | | **Multivariate** | | |
| --- | --- | --- | --- | --- | --- | --- | --- | --- |
|  |  | **N** | **HR** | **CI95%** | **P-value** | **HR** | **CI95%** | **P-value** |
| BCG | No BCG | 23597 | 1 | - | - | 1 | - | - |
|  | BCG | 6060 | 0.89 | 0.80-1.00 | 0.050 | 0.81 | 0.71-0.92 | 0.001 |
| T stage | Ta | 20543 | 1 | - | - | 1 | - | - |
|  | Tis | 1043 | 1.07 | 0.86-1.34 | 0.537 | 1.09 | 0.86-1.39 | 0.459 |
|  | T1 | 8071 | 1.27 | 1.16-1.39 | <0.001 | 1.15 | 1.03-1.28 | 0.011 |
|  |  |  | P(overall): <0.001 | | | P(overall): 0.040 | | |
| Grade | G1/LMP/<NA> | 11178 | 1 | - | - | 1 | - | - |
|  | G2 | 11564 | 1.18 | 1.07-1.29 | <0.001 | 1.10 | 1.00-1.21 | 0.059 |
|  | G3-G4/anaplastic | 6915 | 1.38 | 1.24-1.54 | <0.001 | 1.26 | 1.10-1.44 | <0.001 |
|  |  |  | P(overall): <0.001 | | | P(overall): 0.004 | | |
| Sex | M | 22434 | 1 | - | - | 1 | - | - |
|  | F | 7223 | 0.99 | 0.91-1.09 | 0.901 | 1.00 | 0.91-1.10 | 0.927 |
| Age | <=75 | 18028 | 1 | - | - | 1 | - | - |
|  | >75 | 11629 | 4.24 | 3.89-4.62 | <0.001 | 3.76 | 3.44-4.11 | <0.001 |
| CCI | No comorbidity (0) | 16831 | 1 | - | - | 1 | - | - |
|  | Mild comorbidity (1) | 4790 | 1.91 | 1.71-2.13 | <0.001 | 1.59 | 1.42-1.77 | <0.001 |
|  | Intermediate comorbidity (2) | 4748 | 1.82 | 1.62-2.05 | <0.001 | 1.47 | 1.31-1.66 | <0.001 |
|  | Severe comorbidity (>2) | 3288 | 2.90 | 2.55-3.29 | <0.001 | 2.11 | 1.85-2.41 | <0.001 |
|  |  |  | P(overall): <0.001 | | | P(overall): <0.001 | | |
