# Supplementary material for: The association between BCG treatment in patients with bladder cancer and subsequent risk of developing Alzheimer and other dementia.—A Swedish nationwide cohort study from 1997 to 2019
Source: PLoS One. 2023 Dec 14;18(12):e0292174. doi: 10.1371/journal.pone.0292174 (PMC10721016; doi:10.1371/journal.pone.0292174)
Supplement: Attachment — Submitted filename: A rebuttal letter to PLOS ONE.docx [file pone.0292174.s003.docx]

Eskilstuna, 2023-06-04

Dear Professor Mr Chen, Academic Editor PLOS ONE

Thank you for giving us the opportunity to submit a revised draft of our manuscript titled ¨ The association of BCG treatment in patients with bladder cancer and subsequent risk of developing Alzheimer and other dementia. - A Swedish nationwide cohort study from 1997 to 2019.¨ to PLOS ONE. We are appreciated the time and effort that you and the reviewers have dedicated to providing your valuable feedback on our manuscript.

We are grateful to the reviewers for their insightful comments on our paper. We have been able to incorporate changes to reflect most of the suggestions provided by the reviewers. We have highlighted the changes within the manuscript. Here is a point-by-point response to the reviewers’ comments and concerns.

| Reviewers comments and concerns | our responses and changes |
| --- | --- |
| Editor |  |
| General comments |  |
| 1. Please ensure that your manuscript meets PLOS ONE's style requirements | OK |
| 2. We note that the grant information you provided in the ‘Funding Information’ and ‘Financial Disclosure’ sections do not match. | OK, we will mention our financial disclosures as following:  "This study was funded by Center for Clinical Research, Sörmland, Uppsala University and Schmekel funder for Urological research." |
| 3. Thank you for stating the following financial disclosure:  "This study was funded by Center for Clinical Research, Sörmland, Uppsala University and Schmekel funder for Urological research."  Please state what role the funders took in the study.  If the funders had no role, please state: "The funders had no role in study design, data collection and analysis, decision to publish, or preparation of the manuscript." If this statement is not correct you must amend it as needed.  Please include this amended Role of Funder statement in your cover letter; | The funders had no role in study design, data collection and analysis, decision to publish, or preparation of the manuscript |
| 4. In your Data Availability statement, you have not specified where the minimal data set underlying the results described in your manuscript can be found. PLOS defines a study's minimal data set as the underlying data used to reach the conclusions drawn in the manuscript and any additional data required to replicate the reported study findings in their entirety. All PLOS journals require that the minimal data set be made fully available. For more information about our data policy, please see  5. We note that you have stated that you will provide repository information for your data at acceptance. Should your manuscript be accepted for publication, we will hold it until you provide the relevant accession numbers or DOIs necessary to access your data. If you wish to make changes to your Data Availability statement, please describe these changes in your cover letter and we will update your Data Availability statement to reflect the information you provide. | Data used in the present study was extracted from the research database BladderBaSe, which is based on the Swedish National Registry of Urinary Bladder Cancer (SNRUBC) and linkage to several national health-data registers. The data cannot be shared publicly because the individual-level data contain potentially identifying and sensitive patient information and cannot be published due to legislation and ethical review restrictions (https://etikprovningsmyndigheten.se). Use of the data from national health-data registers is further restricted by the Swedish Board of Health and Welfare (https://www.socialstyrelsen.se/en/) and Statistics Sweden (https://www.scb.se/en/) which are Government Agencies providing access to the linked healthcare registers.  The data in in BladderBaSe is partly available in annual reports from the Swedish National Registry of Urinary Bladder Cancer (SNRUBC) and are also accessible online at https://statistik.incanet.se/urinblasecancer/. Researchers can propose and apply for data and studies in BladderBaSe or SNRUBC using a standardized form. After approved application, the project data administrators can upload study-specific files with selected variables to a server for statistical analysis through remote access.  De-identified data can be available to researchers after application to the BladderBaSe Steering Committee. |
| 6. Please amend either the title on the online submission form (via Edit Submission) or the title in the manuscript so that they are identical. | OK |
| 7. Please upload a copy of Supporting Information Table S1 which you refer to in your text on pages 13 and 21. | OK |
| **Additional Editor Comments:** |  |
| Reviewer #1: |  |
| 1. Median follow-up was 4.6 years, as compared to 8 years in a previous study (ref#7). Since the median patient’s age was 72 years in this study, there was probably not enough time to develop dementia and to make the difference apparent (median age at diagnosis of Alzheimer’s diseases in ref#7 was 84 years). The authors may study a subgroup of their patients in which follow-up is longer. | It is correct that we had a shorter follow-up but the mean age was three years more in our study group. To analyse if the shorter follow-up had an impact we established one subgroup who were 85 years or older at follow-up. As far as we can see, our conclusions will not be changed even in this subgroup.  This information is now added in the results. |
| 2. 6.1% of the study group developed dementia but only 1.5% Alzheimer’s disease. Alzheimer’s disease is responsible for 60-70% of all dementia cases. How can this be explained? | Our data from register was like as we presented, which may be because doctors (especially general practitioners from Health center) did not give detail diagnosis with Alzheimers, but only generally diagnosis with dementia. In discussion, we mentioned: The diagnosis in Sweden is often made by the GP ( general practitioners) and misclassification between dementia subtypes is quite common according to a validation study of the Swedish registry. |
| 3. Page 15, second paragraph, third line should be ref#7. | Ok |
| Reviewer #2: |  |
| 1- Please provide a list of abbreviations, because some abbreviations are mentioned without their full names. | **List of abbreviations:**  AD – Alzheimer´s disease  BCG - Bacillus Calmette-Guerin  BladderBaSe - Bladder Cancer Data Base Sweden  CI – confidence interval  G1/LMP: Bladder cancer Grade 1 or Low Malignant Potential  GP - general practitioner  HR – hazard ratio  ICD – International classification of diseases  NMIBC - non-muscle invasive bladder cancer  Q1-Q3; Inter quartile range, the range between the first quartile (Q1) and the third (Q3)  SNRUBC – Swedish national register of urinary bladder cancer |
| 2- It has been shown that adult vaccination against several infections has reduced the risk of developing Alzheimer (AD); how is the difference in BCG vaccine instillations in patients with NMIBC? | We do not fully understand this question but it is clear that BCG bladder instillations leads to a systemic immune response with for example fever in some patients and getting a positive PPD skin test. |
| 3- Accumulating data argue for the beneficial role of BCG to reduce the risk for developing AD. BCG vaccines are manufactured under different conditions across the globe generating divergent formulations, contributing to differences in the ability of these diverse formulations to induce specific and heterologous protection. Could this strains variation explain your results and conclusions that the BCG treatment is not associated with a significant decreased risk of developing Alzheimer but a marginally lower risk for all dementia diseases? | Yes, using different BCG strains could be one of many factors to influence our results, which we will mention in our discussion. |
| 4- In the perspective of this study would it be possible to evaluate if there is any difference in doses exposure to BCG and risk of developing AD or Dementia? | In discussion we wrote that one limitation was that dose and duration of the treatment, was data we did not have available. |
| 6. PLOS authors have the option to publish the peer review history of their article ([what does this mean?](https://journals.plos.org/plosone/s/editorial-and-peer-review-process#loc-peer-review-history)). If published, this will include your full peer review and any attached files. | OK. |

We look forward to hearing from you in due time regarding our submission and to respond to any further questions and comments you may have.

Sincerely,

Eugen Wang, M.D., Ph.D.

Center for Clinical Research, Region Sörmland, Uppsala University

Sweden
