# Supplementary material for: The association between BCG treatment in patients with bladder cancer and subsequent risk of developing Alzheimer and other dementia.—A Swedish nationwide cohort study from 1997 to 2019
Source: PLoS One. 2023 Dec 14;18(12):e0292174. doi: 10.1371/journal.pone.0292174 (PMC10721016; doi:10.1371/journal.pone.0292174)
Supplement: Attachment — Submitted filename: rebuttal letter to PLOS ONE (2) (002).docx [file pone.0292174.s004.docx]

Eskilstuna, 2023-07-28

Dear Professor Mr Chen, Academic Editor PLOS ONE

Thank you for giving us second opportunity to submit a revised draft of our manuscript titled ¨ The association of BCG treatment in patients with bladder cancer and subsequent risk of developing Alzheimer and other dementia. A Swedish nationwide cohort study from 1997 to 2019.¨ to PLOS ONE. Changes are redmarked.
We appreciate the time and effort that you and the reviewers have dedicated to providing your valuable feedback on our manuscript.

Following is our answer for comment from reviewer 1:

Reviewers comments and concerns*:*

*Previous studies with long follow-up showed significant decrease in the risk of Alzheimer's disease in BCG treated patients. The current study with a short follow-up found only a marginal decrease. One may figure that with longer follow-up the marginal benefit will be more pronounced.*

Our answer:

The median follow-up was 8 years in one study (ref 7) and about 3 years in the other previous studies (ref 5 and 6) compared to 4.6 years in our study. Both these later studies showed a substantial decrease in risk as opposed to our marginal decrease despite similar follow-up length.

To further address the reviewers concern regarding the follow-up time we conducted a subgroup analysis with subjects followed until using the number of years from year of diagnosis to 2019. If this number plus the age of the patient is at least 85, the patients are kept in the analysis. I.e., only patients who could be under risk at age 85 or older are kept in the analysis. Then 12798 patients with a mean age of 79.8 at diagnosis remain with a median follow-up time of about 5 years. The HR was then 0.87 and 0.81 for AD and dementia respectively. This was in the same range as for the whole study group (0.89 and 0.88) with shorter follow up. As more details to explain this point, we will add one more table (Table S2) in support information.

## Table S2 supplement: Subgroup analyses of patients follow-up in relevant age

### Alzheimer’s

Median follow-up time: 5.1 years

|  |  |  | **Univariate** | | | **Multivariate** | | |
| --- | --- | --- | --- | --- | --- | --- | --- | --- |
|  |  | **N** | **HR** | **CI95%** | **P-value** | **HR** | **CI95%** | **P-value** |
| BCG | No BCG | 10804 | 1 | - | - | 1 | - | - |
|  | BCG | 1994 | 0.86 | 0.64-1.17 | 0.339 | 0.87 | 0.62-1.23 | 0.430 |
| T stage | Ta | 8260 | 1 | - | - | 1 | - | - |
|  | Tis | 452 | 0.64 | 0.33-1.24 | 0.181 | 0.70 | 0.35-1.41 | 0.321 |
|  | T1 | 4086 | 1.06 | 0.85-1.33 | 0.615 | 1.07 | 0.83-1.39 | 0.587 |
|  |  |  | P(overall): 0.285 | | | P(overall): 0.426 | | |
| Grade | G1/LMP/<NA> | 4185 | 1 | - | - | 1 | - | - |
|  | G2 | 5375 | 1.06 | 0.84-1.33 | 0.616 | 1.07 | 0.84-1.36 | 0.585 |
|  | G3-G4/anaplastic | 3238 | 1.01 | 0.76-1.34 | 0.953 | 1.05 | 0.74-1.48 | 0.793 |
|  |  |  | P(overall): 0.866 | | | P(overall): 0.862 | | |
| Sex | M | 9654 | 1 | - | - | 1 | - | - |
|  | F | 3144 | 0.96 | 0.76-1.21 | 0.720 | 0.95 | 0.75-1.21 | 0.697 |
| Age | <=75 | 3326 | 1 | - | - | 1 | - | - |
|  | >75 | 9472 | 1.69 | 1.34-2.13 | <0.001 | 1.65 | 1.31-2.09 | <0.001 |
| CCI | No comorbidity (0) | 6010 | 1 | - | - | 1 | - | - |
|  | Mild comorbidity (1) | 2489 | 1.25 | 0.95-1.65 | 0.104 | 1.22 | 0.93-1.60 | 0.159 |
|  | Intermediate comorbidity (2) | 2387 | 1.05 | 0.77-1.43 | 0.781 | 1.00 | 0.73-1.36 | 0.985 |
|  | Severe comorbidity (>2) | 1912 | 1.71 | 1.25-2.35 | <0.001 | 1.61 | 1.17-2.21 | 0.004 |
|  |  |  | P(overall): 0.011 | | | P(overall): 0.029 | | |

### Dementia

Median follow-up time: 4.9 years

|  |  |  | **Univariate** | | | **Multivariate** | | |
| --- | --- | --- | --- | --- | --- | --- | --- | --- |
|  |  | **N** | **HR** | **CI95%** | **P-value** | **HR** | **CI95%** | **P-value** |
| BCG | No BCG | 10804 | 1 | - | - | 1 | - | - |
|  | BCG | 1994 | 0.91 | 0.79-1.05 | 0.191 | 0.81 | 0.69-0.95 | 0.010 |
| T stage | Ta | 8260 | 1 | - | - | 1 | - | - |
|  | Tis | 452 | 0.87 | 0.67-1.15 | 0.335 | 0.93 | 0.70-1.24 | 0.627 |
|  | T1 | 4086 | 1.14 | 1.03-1.27 | 0.015 | 1.11 | 0.98-1.26 | 0.087 |
|  |  |  | P(overall): 0.024 | | | P(overall): 0.153 | | |
| Grade | G1/LMP/<NA> | 4185 | 1 | - | - | 1 | - | - |
|  | G2 | 5375 | 1.00 | 0.89-1.12 | 0.975 | 1.02 | 0.91-1.15 | 0.690 |
|  | G3-G4/anaplastic | 3238 | 1.17 | 1.03-1.33 | 0.017 | 1.21 | 1.04-1.42 | 0.017 |
|  |  |  | P(overall): 0.028 | | | P(overall): 0.039 | | |
| Sex | M | 9654 | 1 | - | - | 1 | - | - |
|  | F | 3144 | 1.01 | 0.90-1.12 | 0.909 | 1.00 | 0.90-1.12 | 0.979 |
| Age | <=75 | 3326 | 1 | - | - | 1 | - | - |
|  | >75 | 9472 | 2.38 | 2.12-2.68 | <0.001 | 2.30 | 2.05-2.59 | <0.001 |
| CCI | No comorbidity (0) | 6010 | 1 | - | - | 1 | - | - |
|  | Mild comorbidity (1) | 2489 | 1.31 | 1.15-1.50 | <0.001 | 1.26 | 1.10-1.44 | <0.001 |
|  | Intermediate comorbidity (2) | 2387 | 1.29 | 1.12-1.49 | <0.001 | 1.20 | 1.05-1.38 | 0.010 |
|  | Severe comorbidity (>2) | 1912 | 1.89 | 1.63-2.20 | <0.001 | 1.74 | 1.49-2.02 | <0.001 |
|  |  |  | P(overall): <0.001 | | | P(overall): <0.001 | | |

Reviewers comments and concerns*:*

*The explanation why only 1.5% patient were diagnosed with Alzheimer's disease (out of 6.1% patients with dementia), due to the poor capacity of the doctors, cast shadow on all their diagnoses.*

Our Answer:

The first referred study (ref 5) presented results separately for AD and other dementia. The AD represented 24% of all dementias as opposed to 25 % in our study. The second referred study (ref 6) reported 6 % with AD as sole diagnosis and 36 % with AD coupled to other types of dementia. The third study (ref 7) did not analyse other dementia than AD.

Reviewer 2 had no comments.

We look forward to hearing from you in due time regarding our submission and to respond to any further questions and comments you may have.

Sincerely,

Eugen Wang, M.D., Ph.D.

Center for Clinical Research, Region Sörmland, Uppsala University

Sweden
