# Supplementary material for: The association between BCG treatment in patients with bladder cancer and subsequent risk of developing Alzheimer and other dementia.—A Swedish nationwide cohort study from 1997 to 2019
Source: PLoS One. 2023 Dec 14;18(12):e0292174. doi: 10.1371/journal.pone.0292174 (PMC10721016; doi:10.1371/journal.pone.0292174)
Supplement: Attachment — Submitted filename: A rebuttal letter to PLOS ONE (3).docx [file pone.0292174.s005.docx]

Eskilstuna, 2023-09-02

Dear Professor Mr Chen, Academic Editor PLOS ONE

Thank you for giving us third opportunity to submit a revised draft of our manuscript titled ¨ The association of BCG treatment in patients with bladder cancer and subsequent risk of developing Alzheimer and other dementia. A Swedish nationwide cohort study from 1997 to 2019.¨ to PLOS ONE. We are appreciated the time and effort that you and the reviewers have dedicated to providing your valuable feedback on our manuscript.

According to the recommendation from reviewer #2, the paper has been now reviewed by an English native speaker, who made changes to a text without altering its meaning.

We look forward to hearing from you in due time regarding our submission and to respond to any further questions and comments you may have.

Sincerely,

Eugen Wang, M.D., Ph.D.

Center for Clinical Research, Region Sörmland, Uppsala University

Sweden
